# Loosening Neuro-Optic Structures Dosimetric Constraints Provides High 5-Year Local Recurrence-Free Survival With Acceptable Toxicity in T4 Nasopharyngeal Carcinoma Patients Treated With Intensity-Modulated Radiotherapy

**DOI:** 10.3389/fonc.2021.598320

**Published:** 2021-02-22

**Authors:** Tingting Zhang, Meng Xu, Jinglin Mi, Hui Yang, Zhengchun Liu, Lulu Huang, Kai Hu, Rensheng Wang

**Affiliations:** ^1^ Department of Radiation Oncology, The First Affiliated Hospital of Guangxi Medical University, Nanning, China; ^2^ Department of Oncology, Liuzhou Worker Hospital, Liuzhou, China; ^3^ Department of Radiotherapy, Affiliated Hospital of Guilin Medical University, Guilin, China

**Keywords:** neuro-optic structures, dosimetric constraints, radiation-induced optic neuropathy, nasopharyngeal carcinoma, intensity-modulated radiotherapy

## Abstract

**Objective:**

Whether the original dosimetric constraints of neuro-optic structures (NOS) are appropriate for patients with nasopharyngeal carcinoma (NPC) undergoing intensity-modulated radiotherapy (IMRT) remains controversial. The present study compared the survival rates and radiation-induced optic neuropathy (RION) occurrence between T4 NPC patients whose NOS were irradiated with a near maximum dose received by 2% of the volume (D2%) >55 Gy and ≤55 Gy. Moreover, the NOS dosimetric parameters and their correlation with RION occurrence were also evaluated.

**Methods:**

In this retrospective study, 256 T4 NPC patients treated with IMRT between May 2009 and December 2013 were included. Patient characteristics, survival rates, dosimetric parameters, and RION incidence were compared between the D2% ≤55 Gy and D2% >55 Gy groups.

**Results:**

The median follow-up durations were 87 and 83 months for patients in the D2% >55 Gy and D2% ≤55 Gy groups, respectively. The 5-year local recurrence-free survival rates were 92.0 and 84.0% in the D2% >55 Gy and D2% ≤55 Gy groups (P = 0.043), respectively. There was no significant difference in the 5-year overall survival (OS) between both groups (D2% >55 Gy, 81.6%; D2% ≤55 Gy, 79.4%; P = 0.586). No patients developed severe RION (Grades 3–5), and there was no significant difference (P = 0.958) in the incidence of RION between the two groups. The maximum dose of NOS significantly affected the RION incidence, with a cutoff point of 70.77 Gy.

**Conclusion:**

Appropriately loosening NOS dosimetric constraints in order to ensure a more sufficient dose to the target volume can provide a better 5-year local recurrence-free survival and acceptable neuro-optic toxicity in T4 NPC patients undergoing IMRT.

## Introduction

Nasopharyngeal carcinoma (NPC) differs from other head and neck carcinomas in that it has a specific geographic distribution, with a peak incidence of 50 cases per 100,000 people in Southeast Asia and Southern China (1). Radiotherapy is the main treatment for non-metastatic NPC because of its anatomical location and sensitivity to radiation (2). Remarkably, the first diagnosis of NPC usually occurs at an advanced stage because the clinical symptoms are atypical and hardly detected (3). Locoregionally advanced NPCs often infiltrate important areas, including the skull base, the cavernous sinus, the orbit, and the neuro-optic structures (optic nerve and optic chiasm; NOS) (4). Dose restriction of planning target volumes (PTVs) is a clinically common solution to protect organs at risk (OARs) undergoing radiotherapy (5). However, an insufficient dose to the target volume can lead to local recurrence, which is one of the most important causes of radiotherapy failure in NPC treatment (6). Thus, reducing dose to the target volume in order to protect OARs may not be a good choice for NPC treatment.

On the other hand, a satisfactory target volume dose coverage inevitably causes several early or late complications in T4 NPC patients. Radiation-induced optic neuropathy (RION) is one of the most serious complications caused by radiation damage to the NOS. RION causes rapid and painless visual loss in one or both eyes within months to years and adversely affects patients’ quality of life (7). Hence, in the dose constraint criterion of the Radiation Therapy Oncology Group 0225 protocol, the maximum dose to the NOS should not exceed 54 Gy (8). Another study conducted by Parsons et al. reported that a maximum dose (Dmax) <55 Gy resulted in a RION incidence <3%, for a Dmax in the range 55–60 Gy, the observed RION occurrence was in the range 3–7%, while a Dmax >60 Gy resulted in a RION incidence of 7–20% (9). In addition, Mayo et al. proposed an increase in TD5/5 to 55 Gy in a quantitative study of clinical normal tissue effects (10). Hoppe et al. also confirmed that when the dose of the NOS is <55 Gy, the incidence of RION is very low (11). However, it is not known whether this dosimetric constraint is suitable for T4 NPC patients treated with intensity-modulated radiotherapy (IMRT).

As proposed in the International Committee of Radiation Units (ICRU) Report 83, D2% was recommended instead of Dmax (12). In this study, the T4 NPC patients undergoing IMRT were divided into the D2% >55 Gy and D2% ≤55 Gy groups to explore whether the dosimetric constraint (D2% ≤ 55 Gy) of NOS is suitable. We compared survival outcomes and RION occurrence between these two groups and investigated the dosimetric predictors of RION in T4 NPC patients after IMRT treatment. Considering the Dmax is still an important evaluation index for tandem organs in clinical practice (13, 14), it was also included in the analysis.

## Materials and Methods

### Study Design and Participants

This study included 256 T4 NPC patients who underwent IMRT treatment between May 2009 and December 2013 at three general hospitals in the Guangxi Zhuang Autonomous Region (the First Affiliated Hospital of Guangxi Medical University, Liuzhou Worker Hospital, and Affiliated Hospital of Guilin Medical University). The inclusion criteria were as follows: histological confirmation of NPC without distant metastasis; no history of radiotherapy, chemotherapy, or surgery; an Eastern Cooperative Oncology Group performance status between 0 and 2; and complete clinical data. All patients had been recently diagnosed by nasopharyngeal biopsy and were staged as T4 (except those only with tumor invasion to the hypopharynx and/or the infratemporal fossa/masticator space) according to the seventh edition of the International Union against Cancer/American Joint Committee on Cancer staging system. None of the patients had visual impairment due to NOS injury, distant metastasis, previous malignancy, or other concomitant malignancies. All the patients were divided into two groups: 125 patients had their NOS irradiated with a D2% greater than 55 Gy (D2% >55 Gy group), and 131 patients had their NOS irradiated with a D2% lower than 55 Gy (D2% ≤55 Gy group). All patients were followed up for >12 months.

### Radiotherapy

Patients were immobilized in the supine position with a head, neck, and shoulder thermoplastic mask. All patients were scanned using computed tomography (CT) with 3-mm serial slices from the cranial roof to the sternoclavicular junction. CT and magnetic resonance imaging (MRI) data were imported into the treatment planning system. The IMRT plans were inversely planned with nine fields of 6-MV photon beams using the Eclipse system. A sliding-window technique using Varian linear accelerators with a Millennium multileaf collimator (Varian Medical Systems, Palo Alto, CA) and analytical anisotropic algorithm dose calculation were used.

**Figure 1:**
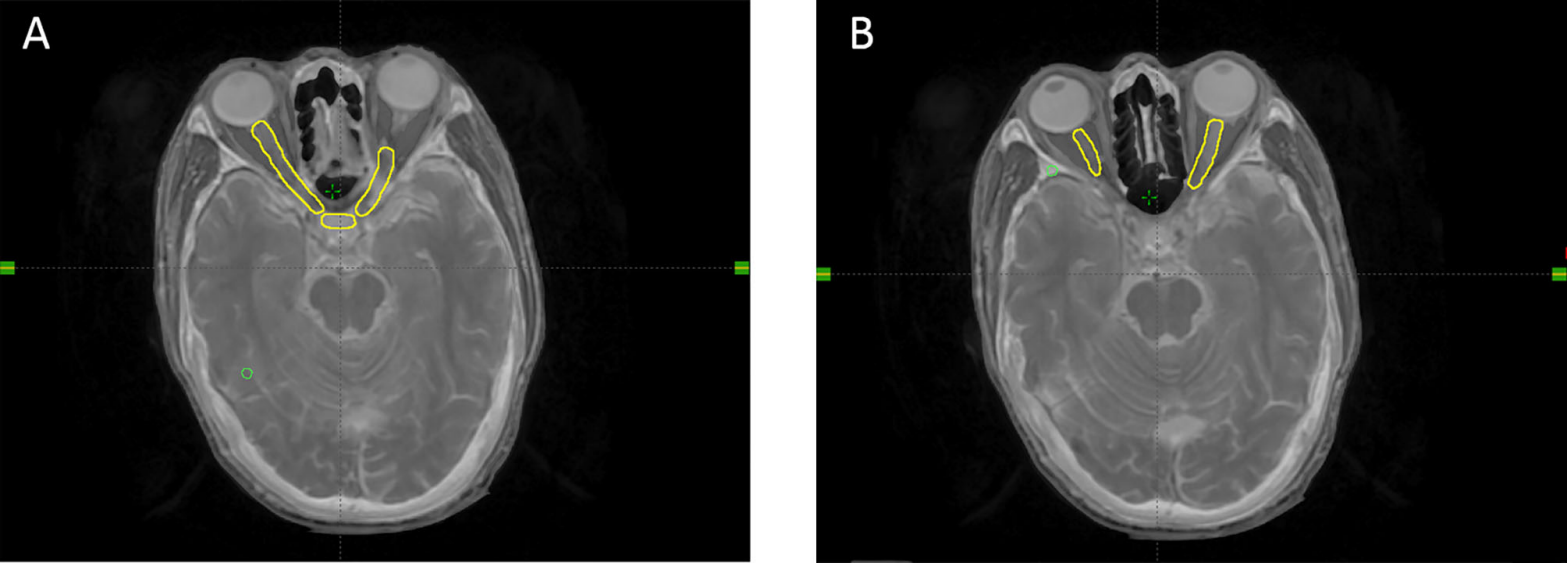
An example of neuro-optic structures **(A, B)**.

Based on the CT and MRI fusion images, the target volumes were designed according to our institutional treatment protocol and reports 50, 62, and 83 of the International Commission on Radiation Units and Measurements (15, 16). The OARs were delineated according to the Radiation Therapy Oncology Group consensus guidelines. The planning target volumes (primary nasopharyngeal tumor: PTVnx; involved lymph nodes: PTVnd: target volume 1: PTV1; target volume 2: PTV2) were generated taking into the account organ movement and the daily treatment configuration by adding 3-mm margins to the gross tumor volume, which included the primary nasopharyngeal tumor (GTVnx), gross tumor volume involving lymph nodes, clinical target volume 1, and clinical target volume 2. A 3-mm margin was added around the OARs to define the planning OAR volume. The prescribed doses delivered to PTVnx, PTVnd, PTV1, and PTV2 were 70–72, 66–70, 60–64, and 52–56 Gy, respectively, in 31–33 fractions. The lower neck region was irradiated separately by a total dose of 50–54 Gy at 2.0 Gy per fraction, using an under-neck tangent beam. An example of a NOS is shown in **Figure 1**. On-board kilovoltage cone beam CT was performed once a week to ensure the accurate position and dosage of the target volumes.

The following NOS dose–volume histogram-based dosimetric parameters were collected: Dmax, D2%, the volume percentage receiving at least 55, 60, 65, and 70 Gy (V55, V60, V65, and V70%, respectively). The endpoints of overall survival and local recurrence-free survival (LRFS) were death and local recurrence, respectively.

### Chemotherapy

Chemoradiotherapy has been recommended for T4 NPC patients. In total, cisplatin-based chemotherapy (80 mg/m^2^ cisplatin every 3 weeks for 2–4 cycles) was administered to 248 (96.7%) of the 256 patients. In the D2% >55 Gy and D2% ≤55 Gy groups, five and three patients refused chemotherapy, respectively. Other patients received concurrent chemotherapy (CCT), neoadjuvant chemotherapy (NACT), NACT + CCT, and CCT + adjuvant chemotherapy according to their condition.

### Outcomes assessment and diagnostic criteria for RION

RION diagnosis was suggested by the clinical setting of a patient with NPC who had received radiotherapy after an appropriate time period since treatment. The RION diagnostic criterion was the observation of an irreversible optic neuropathy or chiasmal dysfunction (impaired visual function with loss of visual acuity and/or visual field defect) without other apparent causes. The Common Terminology Criteria for Adverse Events version 3.0 for visual impairment grading were used (17). Grade 1 was defined as a symptomatic vision change without compromising visual function. Grade 2 was defined as a symptomatic vision change with slight impairment of visual function, but without interfering with activities of daily living (ADLs). Grade 3 was defined as a symptomatic vision change that interfered with ADLs. Grade 4 was defined as blindness (20/200 or worse). Grade 5 was defined as death. Patients with grades 1–2 were defined as having a mild RION, in which they had a good quality of life. Patients with grades 3–5 were defined as having severe RION, corresponding to a significant negative impact on their ADLs.

### Follow-up

Upon IMRT completion, patients were subsequently followed up every 3 months for the first 2 years, every 6 months for the next 3 years, and then once annually. At each follow-up visit, MRI and ophthalmic examinations were performed. RION latency was measured from the first day of irradiation until the day when it was first observed.

### Statistical Analyses

Continuous variables were compared using an independent samples t-test, and categorical variables were compared using the chi-square or Fisher’s exact test. The Kaplan–Meier method was used to estimate survival rates and evaluate the differences in OS and LRFS between groups. Uni- and multivariate Cox regression models were created to determine the dosimetric factors associated with the incidence of RION. The association between dosimetric data and RION occurrence was tested using a logistic regression model. Receiver operator characteristic (ROC) curve analysis was performed and cut-off values on the ROC curve were determined by Youden’s index. All confidence intervals were reported at 95% confidence level. Positive predictive ability curves were generated. Statistical analyses were performed using IBM SPSS Statistics for Windows, Version 25.0 (Armonk, NY: IBM Corp). P values <0.05 were considered statistically significant.

## Results

### Treatment Outcomes

**Figure 2:**
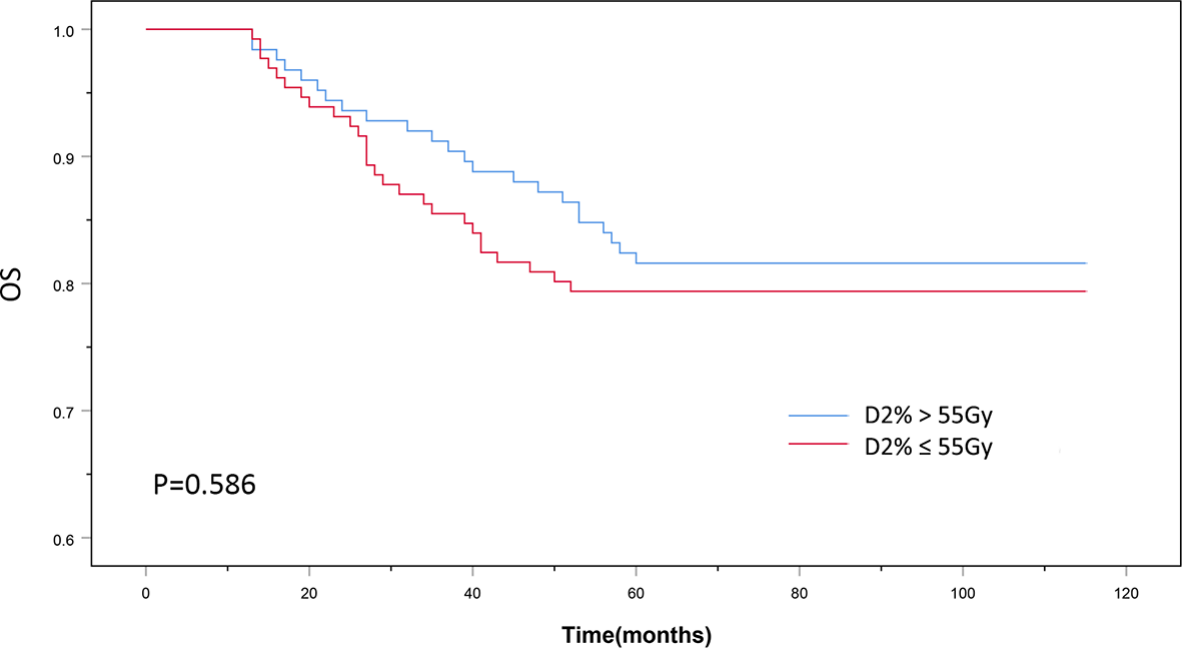
The 5-year overall survival outcomes of D2% >55Gy and D2% ≤55Gy groups treated with intensity-modulated radiotherapy. P < 0.05 was considered statistically significant.

**Figure 3:**
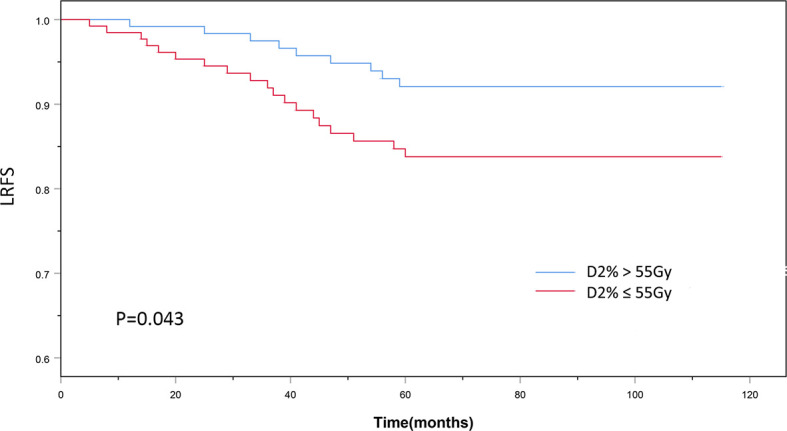
The 5-year local recurrence-free survival outcomes of D2% >55Gy and D2% ≤55 Gy groups treated with intensity-modulated radiotherapy. P < 0.05 was considered statistically significant.

The patients’ baseline characteristics, including age, sex, history of smoking and alcohol consumption, comorbidities, pathology findings, staging, and chemotherapy use, were similar between the two groups (**Table 1**, P > 0.05). Until December 2018, the median follow-up duration was 87 and 83 months in the D2% >55 Gy and D2% ≤55 Gy groups, respectively, with a range from 13 to 115 months. The 5-year OS rates were 81.6 and 79.4% (χ^2^ = 0.297, P = 0.586) (**Figure 2**), while the 5-year LRFS rates were 92.0% and 84.0% (χ^2^ = 4.099, P = 0.043) (**Figure 3**) for patients in the D2% >55 Gy and D2% ≤55 Gy groups, respectively. In the D2% >55 Gy group, during the 5-year follow-up period, 23 (18.4%) patients died and 10 (8.0%) patients developed local failure. In this group, the median time to death and local recurrence was 37 months (range: 13–60) and 41 months (range: 12–59), respectively. In the D2% ≤55 Gy group, 27 (20.6%) patients died and 21 (16.0%) patients developed local failure; the median time to death and local recurrence was 27 months (range: 13–52) and 36 months (range: 5–60), respectively. Local recurrence was observed in the PTV.

**Table 1:** The baseline characteristics of patients.

Characteristics	D2% > 55Gy (n = 125)	D2% ≤ 55Gy (n = 131)	P
**Median age (range)**	45.7 (17–78)	45.2 (13–74)	0.897
**Gender **			0.755
Male	90 (72.0%)	92 (70.2%)	
Female	35 (28.0%)	39 (29.8%)	
**History of smoking**			0.771
Smoker	33 (26.4%)	32 (24.4%)	
Nonsmoker	92 (73.6%)	99 (75.6%)	
**History of alcohol consumption**			0.611
Drinker	19 (15.2%)	23 (17.6%)	
Nondrinker	106 (84.8%)	108 (82.4%)	
**Comorbidity**			0.925
Hypertension	20 (16.0%)	23 (17.6%)	
Diabetes mellitus	7 (5.6%)	8 (6.1%)	
**Histology**			0.85
WHO I	3 (2.4%)	2 (1.5%)	
WHO II	12 (9.6%)	14 (10.7%)	
WHO III	110 (88.0%)	115 (87.8%)	
**N stage**			0.857
N0	10 (8.0%)	15 (11.5%)	
N1	23 (18.4%)	26 (19.8%)	
N2	81 (64.8%)	88 (67.2%)	
N3	1(0.8%)	2 (1.5%)	
**Clinical stage**			0.589
IVa	124 (99.2%)	129 (98.5%)	
IVb	1 (0.8%)	2 (1.5%)	
**Chemotherapy**			0.814
CCT	53 (42.4%)	58 (44.3%)	
NACT+CCT	45 (36.0%)	42 (32.0%)	
CCT+AC	19 (15.2%)	23 (17.6%)	
NACT	3 (2.4%)	5 (3.8%)	
None	5 (4%)	3 (2.3%)	

WHO, World Health Organization; CCT, concurrent chemotherapy; NACT, neoadjuvant chemotherapy; AC, adjuvant chemotherapy; D2%, near maximum dose received by 2% of the volume.

### Dosimetric Data for the Neuro-Optic Structures and the RION Incidence

In the D2% >55 Gy group, the median Dmax was 63.56 Gy (range: 59.95–78.14) and the median D2% was 60.37 Gy (range: 55.05–70.39), while in the D2% ≤55 Gy group, the median Dmax was 57.01 Gy (range: 54.73–63.85) and the median D2% was 53.54 Gy (range: 50.11–54.98). In the D2% >55 Gy group, 16 patients (12.8%) had mild RION, and the median time of NOS toxicity development was 38 months, with a range of 11–86 months. On the other hand, 13 patients (9.9%) had mild optic nerve disorder in the D2% ≤55 Gy group. In this group, the median time interval for NOS toxicity development was 43 months (range: 12–104). There was no significant difference between the two groups (**Table 2**). No patient was diagnosed with severe RION (grades 3–5). In addition, one of the irradiated patients presented with blindness secondary to cataracts. After surgery, the visual acuity was normal. The MRIs of all patients did not show relevant abnormalities, such as enhancement and swelling of the optic nerves or chiasm.

**Table 2:** The incidence of RION.

	Grade	D2% > 55Gy	D2% ≤ 55Gy	
Slight	1	10	8	χ^2^ = 0.003 P = 0.958
	2	6	5	
Serious	3	0	0	
	4	0	0	
	5	0	0	

RION, radiation-induced optic neuropathy; D2%, near maximum dose received by 2% of the volume.

### Dosimetric Factors Associated With RION Occurrence

**Figure 4:**
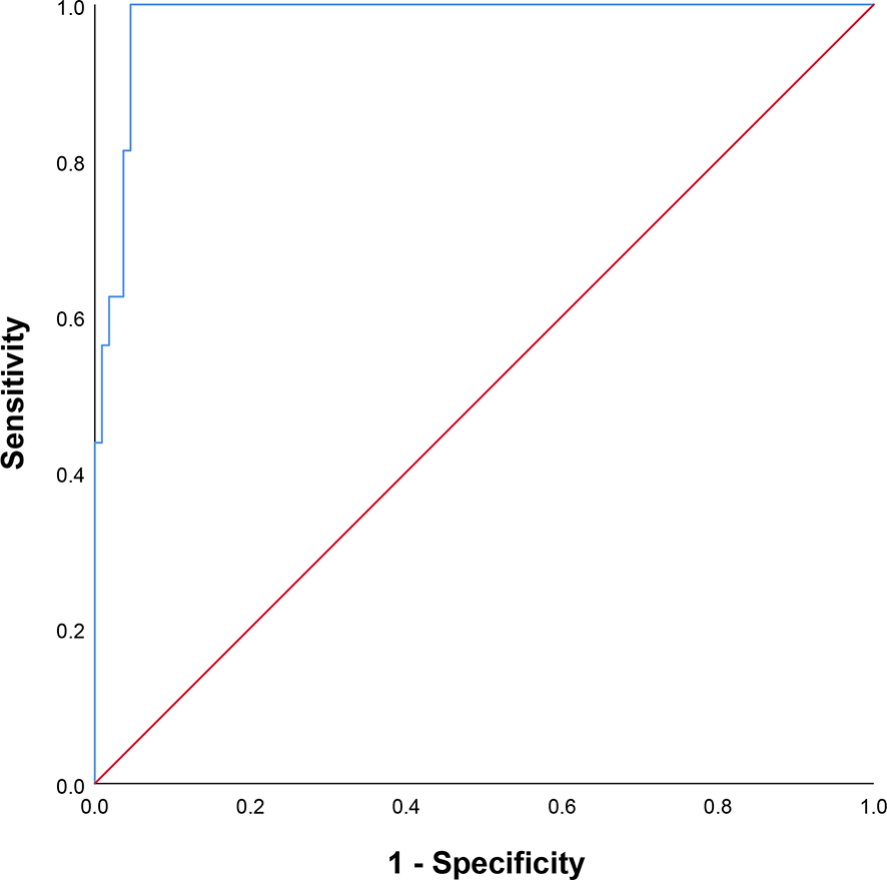
Receiver operating characteristic curve of maximum dose applied to the neuro-optic structures.

**Figure 5:**
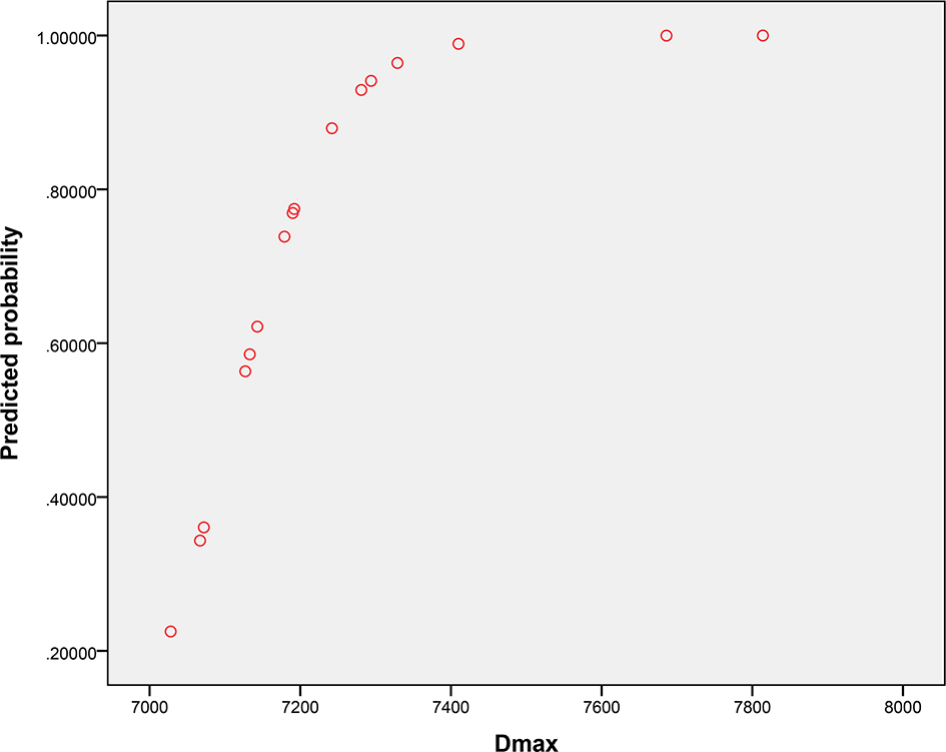
Predictive probability graph of radiation-induced optic neuropathy incidence with increasing maximum doses applied to the neuro-optic structures.

The univariate analysis showed that all dosimetric parameters selected, including V55(%), V60(%), V65(%), V70(%), Dmax, and D2%, were associated with the occurrence of RION (P < 0.05; **Table 3**). However, multivariate Cox regression model revealed that only Dmax was statistically significant and could be identified as an independent predictor of RION (**Table 3**). According to the logistic analysis of the association between dosimetric factors and RION incidence, the odds ratio (OR) attributed to Dmax for RION development was 1.014 (95% confidence interval [CI], 1.002–1.027; P = 0.021). The receiver operating characteristic analysis was used to evaluate the Dmax cutoff point, which was 70.17 Gy (sensitivity 95.4%, specificity 100.0%, Youden’s index 95.4%; AUC = 0.982, P < 0.001, **Figure 4**) for the RION occurrence. The predictive ability graphs showed a linear relationship between Dmax and the risk of developing RION, indicating a tendency for increased RION incidence with increasing Dmax (**Figure 5**).

**Table 3:** Estimated subdistribution hazard ratios for RION using univariate and multivariate cox regression models.

Variables	Univariate analyses		Multivariate analyses	
	Exp (B) (95% CI)	*P*	sHRa (95% CI)	P
V55(%)	1.063 (1.031–1.096)	<0.001		
V60(%)	1.058(1.032–1.084)	<0.001		
V65(%)	1.075(1.038–1.112)	<0.001		
V70(%)	1.19(1.026–1.38)	0.021		
Dmax	1.015(1.006–1.024)	0.001	1.014(1.002–1.027)	0.021
D2%	1.009(1.004–1.014)	0.001		

RION, radiation-induced optic neuropathy; V55(%), volume percentage receiving at least 55Gy; V60(%), volume percentage receiving at least 60Gy; V65(%), volume percentage receiving at least 65Gy; V70(%), volume percentage receiving at least 70Gy; Dmax, the maximum dose; D2%, near maximum dose received by 2% of the volume.

## Discussion

Different studies have reported improved survival and lower incidence of radiation-induced toxicity when using IMRT compared to conventional radiotherapy (18–20). IMRT is widely used for NPC treatment due to achieving local control rates and OS greater than 90 and 80%, respectively (21, 22). Nevertheless, the LRFS rate of T4 NPC patients is much lower than that of patients with NPC in other T stages due to the high tumor load and the tumor proximity to OARs (23). Pan et al. indicated that the 5-year local failure-free survival rate for T4 NPC patients was significantly lower than that for patients with stage T1, T2, and T3 NPC treated with IMRT (P < 0.05) (24). The choice between adequate tumor coverage and reducing the dose delivered to OARs is a challenge for clinicians. The conservative treatment selection may prevent some T4 NPC patients from having a longer LRFS. On the other hand, adequate tumor coverage can lead to good local control. A study carried out by Sun et al. found that high-dose IMRT combined with chemotherapy in locally advanced NPC can improve survival time with low brainstem toxicity (25). A trial by Kwong et al. showed that an increased dose in the target volume showed good local tumor control and increased survival in T3–T4 nasopharyngeal carcinoma (26). In the present study, the 5-year OS was similar between the D2% >55 Gy and ≤55 Gy groups (81.6 *vs*. 79.4%, P = 0.586), but the LRFS rate of the D2% >55 Gy group was significantly higher than that of the D2% ≤55 Gy group (92.0% *vs*. 84.0%, P = 0.043). Therefore, this study indicates that maintaining a high dose to the target volume results in good local tumor control in T4 NPC patients.

Previous studies have suggested that 55 Gy is the tolerance dose for the NOS. As they are close to the nasopharynx, there is a high probability of injury during radiotherapy (6). The dose constraints of NOS should be established in the IMRT treatment, and the oncologist must carefully balance the likelihood of RION and optimal tumor control. In the present study, even though the NOS dosimetric constraint was loosened in the D2% >55Gy group, the occurrence of RION was relatively uncommon; 16 (12.8%) patients had mild RION, and none developed severe RION. This finding is consistent with those of several previous studies. It was reported that 84 patients with sinonasal cancer treated with IMRT using a D2% to the ipsilateral optic nerve, contralateral optic nerve, and optic chiasm of 58.4 ± 5.9 Gy, 51.3 ± 8.6 Gy, and 47.4 ± 10.4 Gy, respectively. However, none of these patients had IMRT-related blindness (Grade 4 ocular toxicity), and only six patients had Grade 3 visual impairment (27). In another dosimetry study, none of the 327 patients with sinonasal cancer who had NOS irradiated with 60 Gy developed RION (28). A study by Dirix showed that in the IMRT treatment mode, the Dmax for the optic chiasm, ipsilateral optic nerve and contralateral optic nerve were 53.3 ± 12.3, 59.6 ± 5.3, and 34.9 ± 14.5 Gy, respectively, and no visual toxicity was reported (29). Daly et al. reported that no patients developed decreased vision when the Dmax for the optic chiasm, ipsilateral optic nerve, and contralateral optic nerve were 52.3 ± 5.1, 59.1 ± 7.7, and 45.2 ± 6.1 Gy, respectively (30). Moreover, Brecht et al. stratified patients into four dose level groups (<50, 50–55, 55–58, and ≥58 Gy) and found no significant differences in the RION incidence between groups (P = 0.494) (31). Therefore, the dosimetric constraint of 55 Gy for the NOS appears to be conservative, and may lead to insufficient target coverage in T4 NPC patients. Despite this promising possibility of exposing NOS to higher doses, maintaining the exposure of the vast majority of nerves and chiasm segments to lower doses may be safe. In this context, it is noteworthy that, unlike conventional radiotherapy, in which a high dose is delivered to NOS targets by bilateral opposed fields, IMRT can deliver a higher radiation dose to the target region while sparing the adjacent optic nerves/chiasm by using multiple fields (32). Thus, only portions of the nerves, rather than the entire nerve, were subjected to the prescription dose at the targeted nerve level.

The fact that a high target dose resulted in a low RION incidence may be partly due to the volume effect in the NOS. A previous study indicated that the incidence of severe ocular toxicity is low in patients receiving a V60% <5% of NOS volume (33). Some trials have indicated that delivering 50–60 Gy to less than 5–30% of the optic nerve volume may reduce the incidence of radiotherapy complications (11, 34). In the report by Martel et al., no cases of RION were found in patients who received average and maximum doses of 53.7 Gy (range: 28–70) and 56.8 Gy (range: 0–80.5), respectively, to the optic chiasm and nerve. However, patients had moderate to severe complications after a Dmax >64 Gy, with 25% of the volume receiving >60 Gy (35). A high single-fraction irradiation dose for a small volume of the anterior visual pathway can be safe and associated with a favorable local tumor control rate located close to the anterior visual pathway structures (36). Another reason for the low RION incidence observed despite the high targeted dose delivered may be that the actual IMRT single-fraction dose to the NOS is usually lower than that of conventional radiotherapy. The OAR tolerance should be reconsidered when the fractionated dose or fractionation times of OARs are significantly reduced, which is different from the recommendations of the ICRU-83 report (37). Reduction of the single-fraction dose can be beneficial for NOS repair (38). The partial volume effect and single-fraction irradiation dose to the NOS have been considered vital determinants of RION development (39).

The radiation tolerance of several other vital intracranial organs has also been investigated by radiation oncologists. Currently, the recommended Dmax for the temporal lobe and brainstem is 60 and 54 Gy, respectively. However, recent studies and clinical experience suggest that the dose tolerance of OARs may be greater than previously reported. A recent study showed that T4 NPC patients treated with IMRT who had a temporal lobe irradiated with a Dmax of 71.14 Gy presented an incidence rate of temporal lobe injury of 12.5% (range: 7.5–28%), which was similar to previous studies (40). Huang et al. showed that patients submitted to a brainstem Dmax <67.4 Gy had a significantly lower risk of developing brainstem injury than those with a Dmax ≥67.4 Gy (OR = 25.29, 95% CI: 8.63–74.14; P < 0.001) (41); indicating that a brainstem Dmax <67.4 Gy can be safe and effective for patients with NPC receiving IMRT treatment. Taken together, these data suggest that the radiation tolerance of these intracranial structures should be reassessed for IMRT treatment planning.

To our knowledge, this is the first study to compare the efficacy and toxicity of a D2% higher and lower than 55 Gy to the NOS of patients with locally advanced NPC. However, there are some limitations to this study that should be noted. This was a retrospective study with data from three hospitals, and potential bias may have occurred. Moreover, in the early era of this century, the target volume dose coverage, conformability, homogeneity, and OARs protection of the IMRT plan for NPC patients were not yet perfect. In addition, the sample size was relatively small, and the conclusions obtained here need to be verified by large-sample prospective studies. Despite these limitations, the results of this study provide evidence that, considering the risk of some invasive head and neck tumor recurrence, the increase in equivalent point doses for the NOS can supply good local tumor control.

In conclusion, appropriately loosening NOS dosimetric constraints in order to ensure a more sufficient dose to the target volume can provide a better 5-year local recurrence-free survival and acceptable neuro-optic toxicity in T4 NPC patients undergoing IMRT. The results presented here suggest that restricting the Dmax to <70.77 Gy during IMRT optimization can significantly reduce the occurrence of RION in T4 NPC patients without compromising tumor dose coverage. To confirm these conclusions, prospective studies based on dose-volume constraints should be performed in the future.

## Data Availability Statement

The raw data supporting the conclusions of this article will be made available by the authors, without undue reservation.

## Ethics Statement

The studies involving human participants were reviewed and approved by The First Affiliated Hospital of Guangxi Medical University. The patients/participants provided their written informed consent to participate in this study. Written informed consent was obtained from the individual(s) for the publication of any potentially identifiable images or data included in this article.

## Author Contributions

TZ, MX, and JM were responsible for methodology, data analysis, and writing and editing of the manuscript. HY, ZL, and LH were responsible for data collections. KH was responsible for modifying the manuscript. RW provided some critical useful suggestions. All authors contributed to the article and approved the submitted version.

## Funding

This work was supported by grants from the International Communication of Guangxi Medical University Graduate Education [30/02103009005X], the Basic Ability Enhancement Project of Young Teachers in Guangxi Zhuang Autonomous Region [2019KY0152], the Research Project of Guangxi Health and Family Planning Commission [Z20180925], the Central Guided Local Science and Technology Development Project [GK ZY18076006], the Guangxi Key Research and Development Plan [GK AD17129013], and the Scientific Research and Technology Development Project [GKH 1599005-2-11].

## Conflict of Interest

The authors declare that the research was conducted in the absence of any commercial or financial relationships that could be construed as a potential conflict of interest.
